# The functions of long noncoding RNAs on regulation of F-box proteins in tumorigenesis and progression

**DOI:** 10.3389/fonc.2022.963617

**Published:** 2022-07-19

**Authors:** Lu Xia, Jingyun Chen, Min Huang, Jie Mei, Min Lin

**Affiliations:** Center for Uterine Cancer Diagnosis and Therapy Research of Zhejiang Province, Department of Obstetrics and Gynecology, The Second Affiliated Hospital of Wenzhou Medical University, Wenzhou, China

**Keywords:** cancer, lncRNAs, F-box protein, treatment, oncogenesis, noncoding RNA

## Abstract

Accumulated evidence has revealed that F-box protein, a subunit of SCF E3 ubiquitin ligase complexes, participates in carcinogenesis and tumor progression *via* targeting its substrates for ubiquitination and degradation. F-box proteins could be regulated by cellular signaling pathways and noncoding RNAs in tumorigenesis. Long noncoding RNA (lncRNA), one type of noncoding RNAs, has been identified to modulate the expression of F-box proteins and contribute to oncogenesis. In this review, we summarize the role and mechanisms of multiple lncRNAs in regulating F-box proteins in tumorigenesis, including lncRNAs SLC7A11-AS1, MT1JP, TUG1, FER1L4, TTN-AS1, CASC2, MALAT1, TINCR, PCGEM1, linc01436, linc00494, GATA6-AS1, and ODIR1. Moreover, we discuss that targeting these lncRNAs could be helpful for treating cancer *via* modulating F-box protein expression. We hope our review can stimulate the research on exploration of molecular insight into how F-box proteins are governed in carcinogenesis. Therefore, modulation of lncRNAs is a potential therapeutic strategy for cancer therapy *via* regulation of F-box proteins.

## Introduction

F-box protein is a subunit in Skp1-Cullin1-F-box protein (SCF) E3 ubiquitin ligase complexes (1). It has been well documented that F-box proteins target their substrates *via* ubiquitination and proteasome degradation (2). It has been accepted that F-box proteins have 69 members in human genome (3). According to specific motifs in F-box proteins, these proteins are classified into three types: 10 FBXW proteins (WD40 repeat domains), and 22 FBXL proteins (leucine-rich repeat motifs), 37 FBXO proteins (other motifs) (4). Accumulated evidence demonstrated that F-box proteins participate in cancer initiation and progression *via* regulation of cell proliferation (5–7), apoptosis (8), motility and metastasis (9), cell cycle (10), EMT (11), cancer stem cells (12, 13), drug resistance (14, 15) and autophagy (16).

Noncoding RNAs have little or no protein coding capacity to encode proteins (17). Based on the lengths of nucleotides, noncoding RNAs are classified multiple types: long noncoding RNA (lncRNA, high than 200 bp), short noncoding RNA (17-30 bp) and mid-size noncoding RNA (31-200 bp) (18). It has been known that microRNA (miRNA) has approximately 22 nucleotides and targets gene expression *via* regulation of post-transcription (19, 20). Now, noncoding RNAs have been validated to critically participate in oncogenesis in various types of cancers (21–25). Not surprisingly, noncoding RNAs regulated numerous cellular biological processes and dysregulated noncoding RNAs lead to various diseases, including cancer (26–29). In recent years, accumulated evidence suggests that noncoding RNAs targets the expression of F-box proteins, leading to carcinogenesis and malignant progression. One review has well summarized the role of noncoding RNAs in regulation of F-box proteins in carcinogenesis (30). However, this review mainly described the role of microRNAs in governing the expression of F-box proteins. Here, we summarized the functions and mechanisms of lncRNAs in controlling F-box protein expression, leading to tumor development and progression.

## LncRNAs regulate the expression of F-BOX proteins

### Targeting FBXW family by lncRNAs

#### LncRNA SLC7A11-AS1 regulates β-TrCP1

SLC7A11-AS1 was downregulated in tumor tissues in patients with gastric cancer, and correlated with poor prognosis in these patients (31). Depletion of SLC7A11-AS1 contributed to tumor growth in cells and in mice *via* controlling the ASK1/p38/JNK signaling pathway in gastric cancer (31). In lung cancer cells, SLC7A11-AS1 facilitated tumor progression *via* suppressing miR-4775 and increasing the expression of TRAIP (32). SLC7A11-AS1 has been revealed to play a key role in chemoresistance in various types of cancers (33). Luo et al. found that SLC7A11-AS1 targeted miR-33a-5p and changed the expression of xCT, weakened cell growth, promoted ROS levels, and regulated cisplatin resistance in gastric cancer (34).

SLC7A11-AS1 was found to be significantly increased in PDAC tissues (35). PDAC cells with gemcitabine resistance have a high expression of SLC7A11-AS1, indicating that SLC7A11-AS1 could play an essential role in regulation of drug resistance. In fact, downregulation of SLC7A11-AS1 potentiated gemcitabine sensitivity in resistant PDAC cells and inhibited the PDAC stemness. In line with this, overexpression of SLC7A11-AS1 increased gemcitabine resistance *via* suppressing intracellular ROS levels by maintaining NRF2 stability (35). Mechanically, SLC7A11-AS1 can bind to the F-box motif of β-TrCP1 (also known as FBXW1), which blocks the ubiquitination and degradation of NRF2. Therefore, SLC7A11-AS1 attenuated β-TrCP-mediated degradation of NRF2, reduced ROS levels, and increased cancer stemness, which promoted gemcitabine resistance in PDAC (35). Hence, targeting SLC7A11-AS1 could overcome gemcitabine resistance to improve treatment benefits in PDAC patients.

#### LncRNA PCGEM1 regulates β-TrcP2

β-TrcP2, also known as FBXW11, has been characterized to take part in tumorigenesis (36). FBXW11 activated the β-catenin/TCF and NF-kappa B pathways and increased cell proliferation in lymphocytic leukemia (37). FBXW11 maintained stem-cell-like characters and enhanced liver metastasis *via* governing SIRT1 transcription in colorectal cancer (38). LncRNA PCGEM1 has been found to participate in the initiation and development of a variety of cancers *via* regulating several signaling pathways (39). LncRNA PCGEM1 expression was remarkably increased in cervical cancer specimens, which was associated with FIGO stage, lymph node metastasis, poor survival and distant metastasis in cervical cancer patients (40). PCGEM1 upregulation stimulated proliferation, invasion, migration, and cell cycle process and reduced apoptosis in cervical cancer cells (40). PCGEM1 can work as a ceRNA to sponge miR-182 and suppress its expression, leading to upregulation of FBXW11. Moreover, PCGEM1 can activate the NF-kappa B and β-catenin/TCF pathways, and this activation by PCGEM1 can be abrogated by knockdown of FBXW11 (40). Altogether, PCGEM1 exerted cervical cancer progression *via* modulation of miR-182 and FBXW11.

### Several lncRNAs regulates FBXW7 expression

F-box and WD repeat domain containing 7 (FBXW7) is well studied and acts as one tumor suppressor gene in human carcinogenesis and tumor progression (41–43). One study identified that several lncRNAs are correlated with Fbxw7 deficiency in radiation-mediated thymic lymphoma (44). In mice with Fbxw7 deficiency, microarray dada from radiation-induced thymic lymphomas revealed that 372 lncRNAs are differentially expressed in tumor tissues. Among these lncRNAs, 170 lncRNAs were decreased, while 202 lncRNAs were increased in thymic lymphomas (44). Moreover, these FBXW7-associated lncRNAs were found to participate in DNA repair, cell cycle processes, lymphocyte activation and cell differentiation. Two lncRNAs (lncRNA position: 5119300, 5162836) were observed to be linked to Anxa2, Cecr2, Zeb1 and Zfp438 expressions, whereas one lncRNA (position: 182808654) was decreased and associated with Ampd1, Cd6, Clip1, Dap, Edaradd and Ptk2b (44). Furthermore, lncRNA A_30_P01032978 is correlated with poor disease free survival in patients with breast cancer (44). In this section, we will discuss how the several lncRNAs regulated the expression of FBXW7 in carcinogenesis.

#### LncRNA MT1JP regulates FBXW7

LncRNA MT1JP has been reported to be a tumor suppressor *via* promotion of the translation of p53 by interaction with TIAR (45). In retinoblastoma, MT1JP plays a tumor suppressive role *via* targeting Wnt/β-catenin signaling pathway (46). In breast cancer cells, MT1JP repressed oncogenesis and reversed cisplatin resistance through sponging miR-24-3p and inhibiting the Wnt/β-catenin (47). Consistently, MT1JP exhibited tumor suppressive functions *via* sponging miR-92-3p and targeting miR-214/RUNX3 axis in breast cancer cells (48, 49). In lung cancer cells, MT1JP blocked cell proliferation, migration and invasion through modulation of miR-423-3p/Bim axis (50). In glioma, MT1JP retarded tumor progression *via* competitively binding with miR-24 (51). In osteosarcoma cells, MT1JP was reported to increase the inhibitory function of miR-646 on FGF2 expression (52). In HCC cells, upregulation of MT1JP modulated cell apoptosis and migratory abilities *via* targeting miR-24-3p and regulating AKT, RUNX3 and p21 (53–55). Furthermore, MT1JP regulated miR-24-3p/Bcl2L2 signaling pathway and reduced lenvatinib sensitivity *via* suppression of apoptosis in HCC (56). Moreover, MT1JP upregulation abrogated the PTEN inactivation *via* miR-32 reduction in HCC cells (57).

In intrahepatic cholangiocarcinoma, MT1JP acted as a protective lncRNA *via* regulation of miR-18a-5p and FBP1 (58). MT1JP regulated miR-214-3p/RUNX3 signaling pathway and subsequently inhibited proliferation and migration of gastric cancer (59). Notably, low expression of MT1JP was related with poor prognosis in patients with gastric cancer (60). LncRNA MT1JP was downregulated in gastric cancer tissues compared with adjacent normal tissues (61). Gastric cancer patients had a better prognosis, who often have higher expression of MT1JP. *In vitro* experiment data showed that lncRNA MT1JP upregulation suppressed proliferation, invasion and migration and enhanced apoptosis of gastric cancer cells (61). *In vivo* data revealed that lncRNA MT1JP reduced tumor sizes and tumor metastasis. Mechanistical analysis demonstrated that lncRNA MT1JP sponged miR-92a-3p and upregulated FBXW7 in gastric cancer (61). Rescue experiments exhibited that downregulation of FBXW7 reversed MT1JP-induced inhibition of proliferation, invasion and migration in gastric cancer (61).

#### LncRNA TUG1 regulates FBXW7

Numerous studies have demonstrated the critical role of lncRNA Taurine upregulated gene 1 (TUG1) in cancer initiation and progression. LncRNA TUG1 underlined a tumor promotive property *via* impairing miR-421-mediated suppression of KDM2A and activating the ERK signaling in colorectal cancer cells (62). TUG1 suppressed cancer progression *via* targeting Siglec-15-mediated anti-immune activity in HCC (63). Moreover, TUG1 was reported to sponge miR-328-3p and increase the SRSF9 mRNA expression in HCC cells, leading to promotion of proliferation, invasion and migration (64). Xiu et al. found that TUG1 enhanced tumor malignant progression *via* binding with miR-516b-5p and increasing H6PD expression (65). Xia et al. reported that TUG1 stabilization by IGF2BP2 increased cisplatin resistance *via* targeting autophagy in colorectal cancer (66). One group identified that TUG1 governed the miR-320a/FOXQ1 axis and caused promotion of bladder tumor malignant phenotypes (67).

Sun et al. discovered that TUG1 increased chemoresistance and enhanced cancer stem cell behaviors *via* stabilizing GATA6 protein in colorectal cancer (68). TUG1 targeted AKT/mTOR signaling pathway *via* sponging miR-582-3p, which promoted ovarian cancer malignant behaviors (69). TUG1 sponged miR-29c-3p and upregulated the expression of VEGFA, which facilitated malignant phenotypes in stomach cancer (70). In addition, TUG1 competitively interacted with miR-29a and triggered the expression of IFITM3 in HCC cells (71). TUG1 promoted tumor progression and metastasis *via* modulating miR-140-3p and Annexin A8 axis in bladder cancer cells (72). Zhang et al. reported that TUG1 targeted miR-187-3p and TESC and modulated the NF-kappa B signaling pathway, which governed progression of pituitary adenoma (73). Li group reported that miR-199a-3p/MSI2 signaling pathway was involved in TUG1-mediated promotion of cell migration, invasion and proliferation in Ewing’s sarcoma (74). TUG1 upregulated the expression of XBP1 by sponging miR-498 in ESCC cells, which enhanced tumor metastasis and growth (75). One study revealed that TUG1 upregulated the expression of FBXW7 and induced FBXW7-triggered SIRT1 ubiquitination and degradation (76). Moreover, TUG1 compromised neuronal mitophagy *via* targeting TUG1/FBXW7 axis in cerebral ischemia and reperfusion injury (76). It is necessary to explore whether TUG1 regulates the expression of FBXW7 in carcinogenesis.

#### LncRNA FER1L4 regulates FBXW7

LncRNA Fer-1-like protein 4 (FER1L4) has been discovered to be involved in development of human cancer (77). Xia and colleagues found that FER1L4 knockdown suppressed cell growth and cell cycle progression *via* interacting with miR-372 and upregulating E2F1 expression in gliomas (78). In lung cancer cells, FER1L4 suppressed metastasis and growth and enhanced apoptosis *via* control of PI3K/AKT and p53 signaling pathways (79, 80). In osteosarcoma cells, FER1L4 regulated cell apoptosis and EMT *via* suppression of miR-18a-5p and promotion of SOCS5 and activation of PI3K/AKT pathway (81). In clear cell renal cell carcinoma (ccRCC) tissues, FER1L4 expression is higher than that in adjacent normal tissues (82). High expression of FER1L4 was linked to tumor grade, stage, metastasis and tumor aggressiveness and patient survival (82). In oral squamous cell carcinoma, FER1L4 facilitated tumor progression through regulation of miR-133a-5p/Prx1 axis (83). In colorectal cancer patients, FER1L4 expression levels were downregulated, while RB1 expression was upregulated. FER1L4 expression was associated with RB1 expression in colorectal cancer patients (84).

FER1L4 sponged miR-1273g-3p and increased the expression of PTEN and led to cell cycle arrest and metastasis suppression in colorectal cancer (85). One study showed that downregulation of FER1L4 inhibited the mRNA levels of RB1 in gastric cancer (86). Moreover, FER1L4 reduced cell growth *via* binding with miR-106a-5p and increased the expression of PTEN at both mRNA and protein levels in gastric cancer (87). Similarly, FER1L4 reduced growth, invasion, migration and metastasis by suppressing the Hippo-YAP signaling pathway in gastric cancer (88). Qiao et al. found that FER1L4 repressed cell proliferation and blocked cell cycle at G0/G1 phase as well as enhanced apoptosis *via* upregulation of PTEN in endometrial carcinoma (89). Furthermore, FER1L4 overexpression was correlated with favorable survival outcome in endometrial carcinoma patients (90). Ma et al. reported that FER1L4 decreased cell invasion and growth and promoted cell apoptosis and cell cycle arrest at G0/G1 phase in ESCC cells (91). In HCC cells, overexpression of FER1L4 attenuated cell migration and proliferation, increased apoptosis through targeting PI3K/AKT signaling pathway (92).

In ovarian cancer cells, FER1L4 upregulation reduced paclitaxel tolerance *via* modulation of the MAPK signaling pathway (93). The lower expression of lncRNA FER1L4 was observed in prostate cancer samples compared with normal prostate tissues (94). Early stage of prostate cancer patients had the higher expression of FER1L4 in prostate cancer specimens. Upregulation of FER1L4 decreased proliferation and increased apoptosis in prostate cancer cells *via* sponging miR-92a-3p and upregulating FBXW7 (94). Depletion of FBXW7 abrogated inhibition of cell proliferation caused by upregulation of FER1L4 in prostate cancer cells, indicating that FER1L4 exerted antitumor activities *via* miR-92a-3p/FBXW7 axis (94).

#### LncRNA TTN-AS1 targets FBXW7

LncRNA Titin-antisense RNA1 (TTN-AS1) has been reported to be involved in tumorigenesis in various type cancers, including esophageal squamous cell carcinoma (ESCC), cervical cancer, gastric cancer and lung cancer (95–98). Lin et al. reported that TTN-AS1 worked as an oncogene and was highly expressed in ESCC cells and tumor specimens, and overexpression of TTN-AS1 enhanced ESCC proliferation and metastasis (95). Mechanistically, TTN-AS1 competitively interacted with miR-133b and increased the expression of Snail1, leading to EMT cascade in ESCC cells (95). In addition, TTN-AS1 sponged miR-133b and increased the expression level of FSCN1 and resulted in invasion cascades in ESCC cells (95). Chen et al. reported that TTN-AS1 enhanced growth and metastasis of cervical cancer cells *via* regulation of miR-573/E2F3 axis (96). Dong et al. revealed that TTN-AS1 stimulated gastric cancer development *via* interacting with miR-376b-3p and KLF12 (97). Luo et al. observed that TTN-AS1 contributed to tumor progression *via* modulating PTEN/PI3K/AKT signaling pathway in lung adenocarcinoma (98). Similarly, TTN-AS1 activated cell invasion and migration *via* governing miR-4677-3p/ZEB1 axis in lung adenocarcinoma (99). In prostate cancer cells, TTN-AS1 reduced cell apoptosis and facilitated cell proliferation *via* binding with miR-193a-5p (100).

LncRNA TTN-AS1 sponged miR-134-5p and increased the expression of malignant brain tumor domain containing 1 (MBTD1), contributing to promotion of viability and drug resistance, inhibition of apoptosis in osteosarcoma cells (101). TTN-AS1 interacted with miR-376a-3p and subsequently upregulated KLF15, resulting in promotion of colorectal cancer progression (102). Cui et al. also reported that TTN-AS1 facilitated the cell invasion and growth through activation of miR-497-induced PI3K/AKT/mTOR pathway in colorectal cancer (103). Fang et al. found that TTN-AS1 enhanced invasion, EMT and cell growth *via* governing miR-139-5p/ZEB1 axis and miR-524-5p/RRM2 axis in breast cancer cells (104, 105). One group studied the role of TTN-AS1 in clear cell renal cell carcinoma and found that TTN-AS1 acted as a sponging RNA of miR-195 to increase the expression of cyclin D1 and promote tumor progression (106). It has been reported that lncRNA TTN-AS1 can sponge miR-15b-5p and regulate the expression of FBXW7 in ovarian cancer (107). The low expression of TTN-AS1 was found in ovarian cancer cells and tumor tissues. Upregulation of TTN-AS1 reduced proliferation and colony formation and stimulated apoptosis in ovarian cancer cells (107). Moreover, knockdown of FBXW7 attenuated the functions of TTN-AS1 upregulation on cell behaviors, suggesting that TTN-AS1 exerts its biological behaviors *via* upregulating FBXW7 in ovarian cancer cells (107).

#### LncRNA CASC2 targets FBXW7

LncRNA cancer susceptibility candidate 2 (CASC2) has been reported to serve as a tumor suppressor in carcinogenesis by sponging several miRNAs (108, 109). Upregulation of lncRNA CASC2 attenuated cell viability, induced apoptosis and affected autophagy *via* regulation of miR-19a and NF-kappa B signaling pathway in colon cancer (108). In line with this finding, lncRNA CASC2 enhance apoptosis and autophagy through targeting TRIM16 expression in colon cancer cells (110). CASC2 promoted berberine-mediated cytotoxicity *via* inhibition of Bcl2 in colorectal cancer (111). One group showed that CASC2 overexpression exhibited antitumor activities through sponging miR-24-3p in thyroid cancer (109). Another group reported that CASC2 increased radiotherapy sensitivity *via* sponging miR-155 in papillary thyroid cancer (112). Similarly, lncRNA CASC2 increased irradiation-triggered endoplasmic reticulum stress *via* regulation of PERK signaling pathway in NSCLC cells (113). In pancreatic cancer cells, lncRNA CASC2 increased the expression of PTEN and retarded cell metastasis *via* sponging miR-21 (114).

LncRNA CASC2 overexpression suppressed cell proliferation and tumor growth in mice in hepatocellular carcinoma (HCC) (115, 116). LncRNA CASC2 enhanced apoptosis and suppressed viability *via* targeting miR-24-3p in HCC cells (116). In TNF-related apoptosis-inducing ligand (TRAIL)-resistant HCC cells, CASC2 targeted miR-18a/receptor-interacting serine/threonine-protein kinase 1 (RIPK1) axis and the NF-kappa B pathway, whereas in TRAIL-sensitive cells, CASC2 affected miR-221/caspase-3 and miR-24/caspase-8 (115). In clinical tissues, HCC patients have lower expression of CASC2, which is associated with a poor overall survival rate (115, 117). Sun et al. observed that lncRNA CAS2 reduced cell viability, invasion and migratory activities *via* directly inhibiting miR-183 in HCC cells (118). Wang et al. reported that lncRNA CASC2 inhibited epithelial-mesenchymal transition (EMT) *via* targeting miR-367 and FBXW7 in HCC cells (117). Overexpression of lncRNA CASC2 repressed invasion and migration of HCC cells and suppressed EMT and blocked metastasis *via* sponging miR-367. In addition, FBXW7 was found to be a downstream target of miR-367 in HCC cells (117). Therefore, CASC2 regulates the expression of FBXW7 *via* regulation of miR-367 in HCC cells.

#### LncRNA MALAT1 targets FBXW7

LncRNA metastasis associated lung adenocarcinoma transcript 1 (MALAT1) has been known to be correlated with tumor metastasis in human cancer (119). MALAT1 expression was linked to the WHO grade, tumor size and poor survival in glioma patients (120). MALAT1 depletion increased proliferation of glioma stem cells and inhibited the expression of Nestin and Sox2, two stemness markers (121). MALAT1-mediated cell proliferation promotion was due to activation of ERK/MAPK signaling pathway in glioma cells (121). Han et al. found that MALAT1 downregulated MMP2 and blocked ERK/MAPK signaling pathway as well as exhibited tumor suppressive behaviors in glioma cells (122). Xiang et al. reported that knockdown of MALAT1 induced apoptosis *via* reduction of Cyclin D1 and Myc in U87 and U251 glioma cells (123). Studies showed that MALAT1 inhibited cell apoptosis and increased cell growth and activated autophagy *via* targeting miR-101 and derepressing Rap1B, RAB5A, ATG4D and STMN1 expression in glioma (124, 125).

MALAT1 was identified to recruit FBXW7 to stimulate the degradation of CRY2 and regulate trophoblast invasion and migration (126). MALAT1 was significantly downregulated in glioma samples and associated with tumor grade, tumor size and Karnofsky Performance status in glioma patients (127). MALAT1 repressed viability of glioma cells *via* suppressing miR-155 *in vitro*. Moreover, FBXW7 was identified as a key downstream molecule of miR-155 in glioma cells. Notably, FBXW7 mediated miR-155-triggered oncogenesis in U87 and SHG139 glioma cells. Strikingly, MALAT1 reduced cell viability by upregulation of FBXW7 expression due to downregulation of miR-155 (127). Hence, MALAT1 might be a potential therapeutic target for glioma.

#### LncRNA TINCR targets FBXW7

LncRNA terminal differentiation-induced lncRNA (TINCR) have been implicated in carcinogenesis and tumor progression (128). TINCR can reduce cell invasion and growth, and induce apoptosis through controlling the expression of miR-424-5p and LATS1 in cutaneous malignant melanoma (129). TINCR attenuated cell invasion and growth *via* targeting miR-210 and BTG in laryngeal squamous cell carcinoma (130). In HCC cells, TINCR enhanced cell invasion and growth *via* regulation of STAT3 pathway by binding to TCPTP (131). In breast cancer, TINCR governed cell metastatic ability and cell growth *via* regulating miR-761 and targeting OAS1 and EGFR (132–134).

In lung cancer tissues, TINCR expression levels were downregulated (135). In lung cancer cells, TINCR upregulation retarded cell invasion and proliferation *via* acting as a sponge of miR-544a. Moreover, FBXW7 was validated as a downstream target of miR-544a in lung cancer cells. In a rescue experiment, depletion of FBXW7 abrogated the suppression of TINCR on invasion and proliferation (135). Altogether, lncRNA TINCR performed anti-proliferative and invasive abilities in lung cancer cells through modulating miR-544a/FBXW7 axis. However, one study found that TINCR promoted tumor progression by BRAF-induced MAPK pathway in NSCLC (136). Therefore, further investigation is essential to determine the role of TINCR in lung cancer progression.

#### LncRNA MALAT1 targets FBXW8

MALAT1 has been validated to have a role in cancer diagnosis, prognosis and therapy (137). Emerging study has shown that MALAT1 can regulate the expression of FBXW8 in human cancer (138). FBXW8 has been reported to involve in cell growth and cell cycle progression in choriocarcinoma (139). Depletion of FBXW8 by siRNA transfection suppressed cell growth and induced cell cycle arrest at G2/M phase in choriocarcinoma JEG-3 cells (139). Overexpression of FBXW8 exhibited the opposite functions on cell growth and cell cycle. FBXW8 can regulate the expression of CDK1, CDK2, p27, Cyclin A and Cyclin B1 in choriocarcinoma cells (139). One study reported that miR-218 suppressed the cell proliferation *via* inhibition of FBXW8 in choriocarcinoma JEG-3 cells (140).

In choriocarcinoma cells, MALAT1 upregulation increased cell proliferation, while depletion of MALAT1 hindered cell growth (138). Moreover, MALAT1 exerted its biological behaviors *via* targeting miR-218 in choriocarcinoma cells. Depletion of MALAT1 reduced the tumor growth *in vivo*. What is more, FBXW8 was found to be a direct target of miR-218 and was involved in MALAT1-meidiated promotion of cell proliferation in choriocarcinoma (138). Hence, MALAT1 promoted cell proliferation *via* interaction with miR-218 and upregulation of FBXW8 in choriocarcinoma.

### Targeting FBXO family by lncRNAs

#### Linc01436 regulates FBXO11

Linc01436 was reported to be controlled by E2F6 and served as a tumor promoter in NSCLC cells (141). Linc01436 worked as a miR-30a-3p sponge to increase the expression of EPAS1 in NSCLC, resulting in promotion of cell growth, invasion and migration *in vitro* and enhancement of tumor growth and tumor metastasis in mice (141).

Emerging evidence has revealed that linc01436 plays an oncogenic role in gastric cancer progression (142–144). Linc01436 repressed the expression of miR-585-3p and increased mitogen-activated protein kinase 1 (MAPK1) expression, which contributed to gastric cancer development (143). Similarly, linc01436 triggered gastric cancer progression through modulation of miR-513a-5p and apurinic/apyrimidinic endodeoxyribonuclease 1 (APE1) (144). The higher expression of linc01436 was observed in tumor tissues of gastric cancer patients and was associated with a poor survival in gastric cancer cases (142). Moreover, using *in vitro* experiments, knockdown of linc01436 retarded metastasis and blocked proliferation in BGC823 gastric cancer cells, while increased linc01436 promoted metastasis and proliferative activity in AGS gastric cancer cells (142). Mechanistically, miR-585 can bind to linc01463 and FBXO11, suggesting that linc01436 sponges miR-585 and inhibit it, leading to indirect promotion of FBXO11 expression in gastric cancer (142). Taken together, linc01463 targets miR-585/FBXO11 axis and subsequently promotes progression of gastric cancer.

#### LincRNA GATA6-AS1 regulates FBXO11

Xu et al. reported that lincRNA GATA6-AS1 regulated invasive and migratory capacities and viability *via* binding to miR-19a-5p and increasing TET2 in ovarian cancer cells (145). LincRNA GATA6-AS1 promoted GATA6 expression and controlled the behaviors of lung cancer cells (146). In lung cancer cells, lincRNA GATA6-AS1 suppressed cell proliferation and invasive ability (147). Using several approaches, including RNA sequencing dataset, RT-qPCR and TCGA data, one group found that GATA6-AS1 expression levels were downregulated in lung cancer tissues (147). Moreover, GATA6-AS1 overexpression increased the expression of FBXO11 and SP1 *via* sponging miR-324-5p, contributing to enhancement of invasion and proliferation in lung cancer cells. Furthermore, miR-324-5p overexpression abolished the effects of GATA6-AS1 upregulation in lung cancer (147). In a word, lincRNA GATA6-AS1 might regulate miR-324-5p/FBXO11 axis and facilitated lung cancer development.

#### LncRNA ODIR1 regulates FBXO25

FBXO25 has been reported to participate in cancer development and malignant behaviors (148, 149). Impairing PRKCD-FBXO25-HAX-1 signaling pathway led to lymphomagenesis and reduced the apoptotic reaction (150). FBXO25 facilitated cell invasion, migration and proliferation *via* regulation of YAP, cyclins, MMPs and β-catenin in NSCLC cells (148). Clinically, FBXO25 had the higher expression in the nucleus and cytoplasm of tumor tissues in lung cancer patients, and was associated with lymph node metastasis and TNM stage and overall survival (148). In cutaneous squamous cell carcinoma cells, FBXO25 increased cell growth and metastasis *via* binding with Oct-1, a Cyclin D1 repressor, and stabilization of Cyclin D1 (149). One study showed that lncRNA RP11-527N22.2, also known as osteogenic differentiation inhibitory lncRNA 1 (ODIR1), interacted with FBXO25 and promoted the destruction of FBXO25 protein by recruiting Cullin 3 (151). FBXO25 promoted H2BK120 ubiquitination and increased the trimethylation of H3K4 (H3K4me3), which increased osterix transcription and the expression of osteocalcin, osteopontin and ALP (151). In human umbilical cord-derived mesenchymal stem cells, downregulation of ODIR1 contributed to osteogenic differentiation, while upregulation of ODIR1 suppressed osteogenic differentiation (151). It is required to investigate the role of ODIR1-mediated FBXO25 disruption in oncogenesis and progression.

#### Linc00494 regulates FBXO32

FBXO32 promoter hypermethylation has been revealed to be linked to poor prognosis in patients with ovarian cancer (152). FBXO32 has been involved in carcinogenesis and tumor malignant behaviors. FBXO32 worked as an E3 ligase for PHPT1 ubiquitination, leading to reduction of PHPT1 accumulation, inactivation of the ERK/MAPK axis, which inhibited the proliferation of lung cancer cells (153). FBXO32 repressed tumorigenesis by targeting KLF4 for ubiquitination and proteasomal degradation in breast cancer (154). Linc00494 was predicted to bind with NF-kappa B1 by bioinformatics analysis in ovarian cancer cells (155). Dual-luciferase reporter assay, RIP and RNA pull-down confirmed the interaction between linc00494 and NF-kappa B1. Linc00494 increased the activity of NF-kappa B1 after their interaction. Moreover, NF-kappa B1 suppressed the transcription of FBXO32 *via* binding with the promoter region of FBXO32. Linc00494 upregulation accelerated the expression of NF-kappa B1 and caused invasion, migration and tumorigenesis in ovarian cancer cells. In consistent, upregulation of FBXO22 reversed the linc00494-mediated tumorgenicity in ovarian cancer (155). Strikingly, linc00494 expression levels were highly upregulated in ovarian cancer tissues, while FBXO32 has a lower expression in ovarian tumor specimens (155). In summary, linc00494 modulated NF-kappa B1 and FBXO32 and enhanced progression of ovarian cancer.

## Conclusions and future perspectives

**Figure 1:**
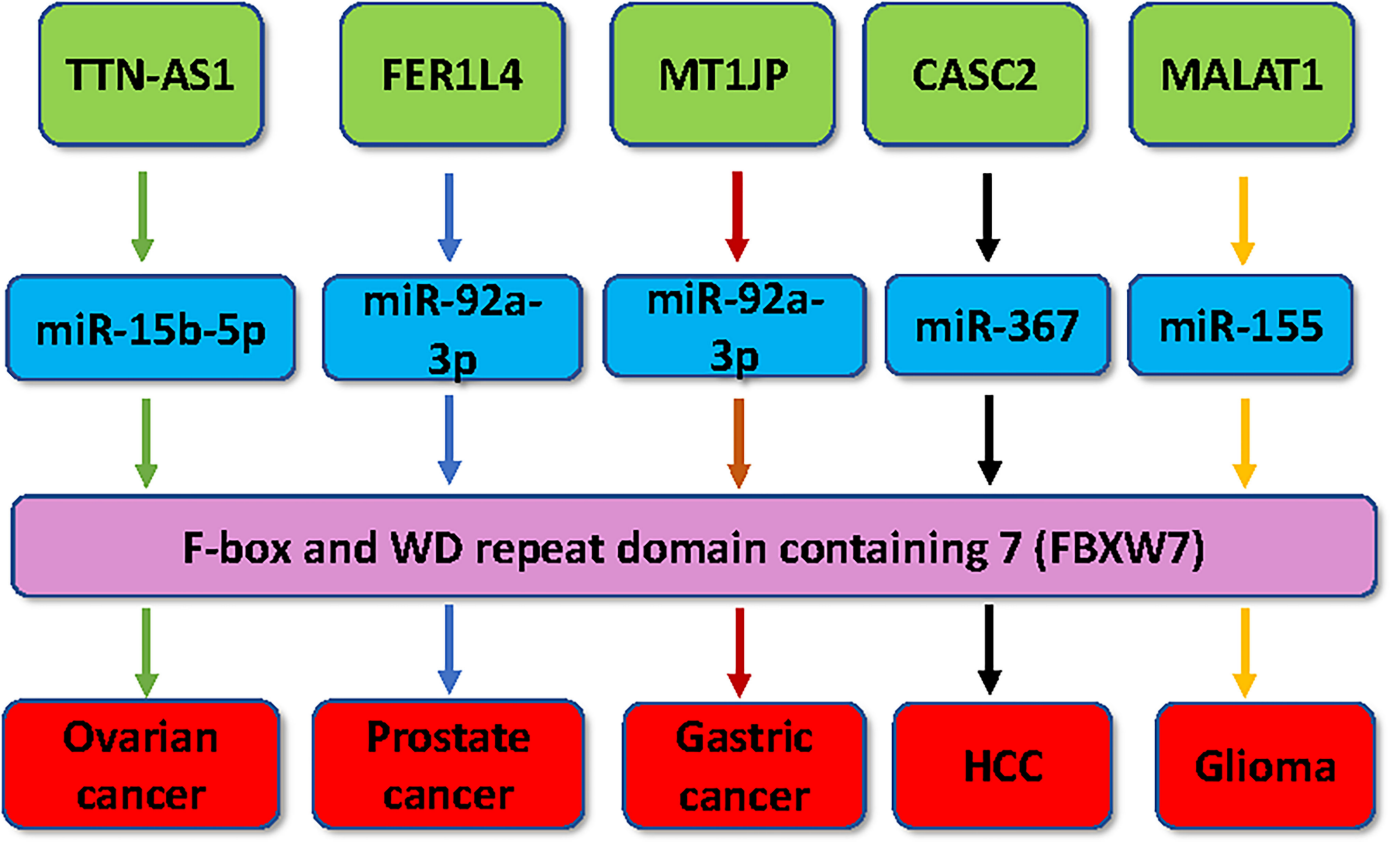
Multiple lncRNAs regulate the expression of FBXW7 in human cancer. Multiple lncRNAs, including MT1JP, FER1L4, TTN-AS1, CASC2 and MALAT1, have been demonstrated to regulate the expression of FBXW7 in tumorigenesis.

**Figure 2:**
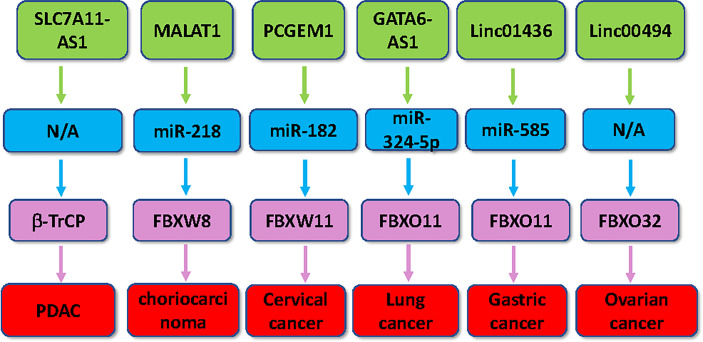
Multiple lncRNAs regulate the expression of F-box proteins in human cancer. Multiple lncRNAs, including SLC7A11-AS1, MALAT1, PCGEM1, linc01436, linc00494 and GATA6-AS1, regulate the expression of several F-box proteins in tumorigenesis.

In conclusion, multiple lncRNAs have been reported to regulate the expression of several F-box proteins in tumorigenesis, including lncRNAs SLC7A11-AS1, MT1JP, TUG1, FER1L4, TTN-AS1, CASC2, MALAT1, TINCR, PCGEM1, linc01436, linc00494, GATA6-AS1, and ODIR1 (**Figures 1** and **2**). Modulation of these lncRNA expressions is a potential therapeutic strategy for cancer therapy *via* regulation of F-box proteins. Besides lncRNAs, miRNAs and circRNAs have also participated in modulation of F-box protein in carcinogenesis. It is necessary to note that several issues need to be addressed for clarifying the functions of lncRNAs in oncogenesis *via* targeting F-box proteins. For example, there are thousands of lncRNAs. However, only about a dozen lncRNAs were identified to regulate the expression of F-box proteins. More lncRNAs should be discovered, which modulate the F-box protein expression in cancer. Among the 69 F-box proteins, no lncRNA was discovered to target FBXL proteins in tumorigenesis. In addition, one lncRNA can target several F-box proteins. For example, MALAT1 targets both FBXW7 and FBXW8 in cancer cells. It is unclear whether MALAT1 targets two F-box proteins at the same time in carcinogenesis. Hence, further in-depth investigation is pivotal to determine whether regulation of F-box proteins by related lncRNAs is a therapeutic strategy for cancer treatment.

## Author contributions

LX drafted the original manuscript and made the figure. JC, MH, and JM edited the manuscript. ML edited this manuscript and supervised this study. All authors have read and approved the final manuscript. All authors contributed to the article and approved the submitted version.

## Funding

This work was sponsored by Natural Science Foundation of Zhejiang province (LY22H160033) and Wenzhou Medical University Basic Research Project KYYW202129.

## Conflict of interest

The authors declare that the research was conducted in the absence of any commercial or financial relationships that could be construed as a potential conflict of interest.

## Publisher’s note

All claims expressed in this article are solely those of the authors and do not necessarily represent those of their affiliated organizations, or those of the publisher, the editors and the reviewers. Any product that may be evaluated in this article, or claim that may be made by its manufacturer, is not guaranteed or endorsed by the publisher.

## Abbreviations

CASC2, Cancer Susceptibility 2; FER1L4, Fer-1 Like Family Member 4; GATA6-AS1, GATA6 Antisense RNA 1; HCC, Hepatocellular carcinoma; LncRNA, Long noncoding RNA; MALAT1, Metastasis-associated lung adenocarcinoma transcript 1; MT1JP, Metallothionein 1J, pseudogene; NRF2, Nuclear factor erythroid-2-related factor 2; ODIR1, osteogenic differentiation inhibitory regulator 1; PCGEM1, Prostate cancer gene expression marker 1; PDAC, Pancreatic ductal adenocarcinoma; ROS, Reactive oxygen species; SLC7A11-AS1, Solute carrier family 7 member 11 antisense RNA 1; SCF, Skp1-Cullin1-F-box protein; STAT3, Signal transducer and activator of transcription 3; TCPTP, T-cell protein tyrosine phosphatase; TINCR, Terminal differentiation-induced non-coding RNA; TUG1, Taurine up-regulated 1; TTN-AS1, TTN Antisense RNA 1.
